# Low- and High-Temperature Phenotypic Diversity of *Brassica carinata* Genotypes for Early-Season Growth and Development

**DOI:** 10.3389/fpls.2022.900011

**Published:** 2022-06-14

**Authors:** Leelawattie Persaud, Raju Bheemanahalli, Ramdeo Seepaul, K. Raja Reddy, Bisoondat Macoon

**Affiliations:** ^1^Department of Plant and Soil Sciences, Mississippi State University, Starkville, MS, United States; ^2^North Florida Research and Education Center, University of Florida, Quincy, FL, United States; ^3^United States Department of Agriculture-National Institute of Food and Agriculture, Clinton, MS, United States

**Keywords:** biomass partitioning, cold stress, heat stress, plant vigor, thermal tolerance

## Abstract

Temperature is a major abiotic stress factor limiting plant growth and development during the early developmental stage. Information on carinata (*Brassica carinata* A. Braun) traits response to low and high temperatures is necessary for breeding or selecting genotypes suited for specific ecoregions, which is limited. In the present study, 12 carinata genotypes were evaluated under low (17/09°C), optimum (22/14°C), and high (27/19°C) day/night temperatures at the early developmental stage. This study quantified temperature effects on several physiological and morphological characteristics of 12-advanced carinata lines. High-temperature plants decreased (15%) the accumulation of flavonoids and increased the nitrogen balance index by 25%. Low-temperature treatment significantly inhibited the aboveground (plant height, leaf area, number, and shoot weight) and root (length, surface area, and weight) traits. Across all genotypes, the shoot weight decreased by 55% and the root weight by 49% under low temperature. On the other hand, the maximum proportion of biomass was partitioned to roots under low temperature than at the high temperature. A poor relationship (*r*^2^ = 0.09) was found between low- and high-temperature indices, indicating differences in trait responses and tolerance mechanisms. AX17004 and AX17009 with higher root to shoot ratios might be suitable for late planting windows or regions with low-temperature spells. The two genotypes (AX17015 and AX17005) accumulated higher biomass under low- and high-temperature treatments can be used for planting in later summer or early winter. The identified low- and high-temperature stress-tolerant carinata genotypes could be a valuable resource for increasing stress tolerance during the early developmental stage.

## Introduction

Globally, carinata (*Brassica carinata* A. Braun) is an important oilseed crop in several countries, including the United States (Seepaul et al., 2021). The origin of the oilseed crop makes it well adapted to its native habitat, the highlands of Ethiopia, in cold temperatures of 14–18°C at elevations of 2200–2800 m above sea level. Along with other oilseed species, such as oilseed rape and canola, the use of carinata for biofuel production has picked up interest in recent years due to its high concentration of erucic acid (Escobar et al., 2009). It also has other industrial uses such as manufacturing plastics, lubricants, paints, leather tanning, soaps, and cosmetics (Taylor et al., 2010). Carinata has the potential to reduce weed pressure during the growing season, and it can be used as a feed crop due to its combination of low-fiber and high-protein (Seepaul et al., 2021). This crop has a long growing season of 180 days (Alvarado and Bradford, 2002). The yields of *Brassica* species are highly dependent on environmental conditions during their growth and developmental stages (Nóia Júnior et al., 2022). Carinata can be double-cropped as a winter cover crop in subtropical regions (Kumar et al., 2020). Field tests across Canada and various areas of the United States (Marillia et al., 2014; Mulvaney et al., 2019) established management practices for carinata cultivation (Magarey et al., 2008).

Carinata is a relatively new winter oilseed crop in the southeastern United States (Christ et al., 2020), where studies are currently ongoing to identify lines best suited for commercial production (Kumar et al., 2020) and understand how this crop would fit the local cropping systems in the United States (Mulvaney et al., 2019; Seepaul et al., 2021; Nóia Júnior et al., 2022). To support the adoption and commercialization of carinata production in the southeastern United States, a consortium known as the Southeastern Partnership for Advanced Renewables from Carinata (SPARC), led by the University of Florida (UF), is focused on removing physical, environmental, economic, and social constraints to its adoption and production, and reduce risks along the supply chain (George et al., 2021). Carinata has been cultivated commercially as a summer crop in the Canadian prairie and the northern plains of the United States and as a winter crop in the Southeastern United States (Seepaul et al., 2021). Currently, there is an opportunity for row crop growers in the Southeastern United States to invest in the cultivation of carinata to diversify their existing systems and profitability (Christ et al., 2020; Nóia Júnior et al., 2022). Since the carinata crop is planted in late fall in the United States, variations in late fall (low temperature) or late summer and early spring (high temperature) temperatures can affect growth and developmental events. Variations in temperatures affect the carinata crop establishment and growth when trying to plant it as a rotation crop or cover crop during fall in the United States. For example, carinata planting dates vary by region (North Carolina growers plant their carinata between late September/early October; while Florida growers plant in mid or late November). These stressful events could affect early seedling vigor, canopy growth (leaf area), and root development. It was reported that earlier/late planting is most likely to result in weaker plants (reduced vigor) at the seedling stage due to reduced leaf and root growth and biomass production. To take the fullest advantage of management practices, there is a need to identify carinata genotypes that maintain superior vigor (high biomass) at the early growth stage under low- and high-temperature conditions.

Temperature is an important abiotic stress factor that plays a dominant role in controlling plant growth and developmental processes. Plant species, and genotypes within species, vary in their sensitivity to temperature (Munyon et al., 2021; Reddy et al., 2021). Several studies used variations in morpho-physiological and yield responses to evaluate stress tolerance in oilseed crops: canola (Elferjani and Soolanayakanahally, 2018), peanut (*Arachis hypogaea* L.) (Kakani et al., 2002), and cotton (*Gossypium hirsutum*) (Reddy et al., 2020). In addition, to shoot traits, root traits have been used to investigate crop responses to a range of stresses, including drought (Raju et al., 2014), low temperature (Reddy et al., 2021), high temperature (Alsajri et al., 2019), nutrient (Jia et al., 2022), salinity (Kakar et al., 2019), waterlogging (Walne and Reddy, 2021), and UV-B (Ramamoorthy et al., 2022). These studies identified morpho-physiological traits enabling the selection of superior stress-tolerant genotypes at the early growth stage in rice (Kakar et al., 2019; Reddy et al., 2021), corn (*Zea mays*) (Wijewardana et al., 2015), sweet potato (*Ipomoea batatas*) (Ramamoorthy et al., 2022), and cotton (Brand et al., 2016). Likewise, variations in stress tolerance among carinata genotypes have been reported (Angadi et al., 2000; Gesch et al., 2019; Zoong Lwe et al., 2021). There is limited information on carinata response to different temperature ranges during the early season.

In this study, we hypothesized that introducing early-stage chilling (low)/heat stress (high temperature) tolerance can help reduce the impact of temperature stress on carinata production. One of the ways to minimize the stress (low- and high temperature) effect is by identifying stress-tolerant genotypes at the early vegetative stage. To address the above knowledge gaps, we screened 12 carinata genotypes to identify low and high-temperature stress-tolerant genotypes, one of the prerequisites for breeding for tolerance or expanding the genetic base. The present study was conducted with the following objectives (a) to determine how low and high temperatures affect the early vegetative growth of carinata, (b) to determine which carinata parameters (physiology, shoot, and root growth) are sensitive to low- and high-temperature stress at the early vegetative stage, and (c) to classify carinata genotypes based on a stress response for low and high temperatures stress.

## Materials and Methods

### Experimental Conditions

This study was conducted at the Rodney Foil Plant Science Research facility of Mississippi State University, Mississippi State, MS (33°20′N, 88°47′W), from November to December 2018. Carinata genotypes were planted in three sunlit, controlled environment units called Soil-Plant-Atmosphere-Research (SPAR) chambers. Each of these chambers consists of a built-in soil bin made from steel (1-m depth × 2-m length × 0.5-m width) to accommodate belowground plant parts and a transparent chamber made of 1.27-cm thick Plexiglas (2.5-m height × 2-m length × 1.5-m width) as room for aboveground plant growth. The Plexiglas on each unit allows 97% of visible incoming solar radiation to pass without spectral variation in absorption, with a wavelength of 400–700 nm (Zhao et al., 2003). These SPAR chambers are equipped to monitor and control air temperature accurately and maintain the atmospheric CO_2_ concentration at a fixed calibrated point. The SPAR chambers are also equipped with a cooling and heating system connected to air ducts that carry conditioned air through the crop canopy to cause leaf flutter. Further details on this SPAR unit control and operations were described by Reddy et al. (2001).

Additionally, chilled ethylene glycol was provided *via* parallel solenoid valves to the cooling system, which opened or closed based on the cooling requirement. Two electrical resistance heaters, which give off short heat pulses to regulate the air temperature, provide the required heat. Humidity and temperature sensors (HMV 70Y, Vaisala Inc., San Jose, CA, United States) installed in the returning path of the airline ducts helped to monitor the relative humidity. Different density of shade cloths placed around the perimeter of the plant canopy designed to simulate canopy spectral properties was readjusted to match the canopy height daily, which also eliminated the need for border plants. The CO_2_ concentration, the air temperature inside the chamber, an irrigation system in each SPAR unit, and the continuous monitoring of plant and environmental gas exchange variables were automatically controlled and monitored every 10 s by a dedicated network system, also equipped to record and store data automatically. Soil moisture was monitored in all SPAR units using soil moisture probes (5TM Soil Moisture and Temperature Sensor, Decagon Devices, Inc., Pullman, WA, United States). These probes were inserted at a depth of 15 cm from the surface of five pots in each temperature treatment and set to measure soil moisture content every 60 s and recorded it at 15-min intervals. The CO_2_ concentration inside the chamber was measured and maintained at 420 μmol mol^–1^ daily. Irrigation was done with installed fertigation systems with a full-strength Hoagland plant nutrient solution. This process was carried out three times daily using an automatic drip system.

### Seed Materials and Temperature Treatments

For this study, seed material of 11 advanced carinata genotypes of 3 breeding types (inbred, double haploid, and hybrid) close to commercial deployment and 1 commercial check genotype were evaluated (Table 1). Seeds sourced from Agrisoma Biosciences Inc., Canada (now Nuseed) were treated with Helix Vibrance, which contains four fungicides (difenoconazole, metalaxyl-M, fludioxonil, and sedaxane) and one insecticide (thiamethoxam), to control insects and diseases. The treated seeds were sown in 180 polyvinyl-chloride (PVC) pots (5.24-cm diameter, 30.5-cm height, and 5.5-L volume) filled with 3:1 sand and soil. The pots were initially sown with four seeds and thinned to one seedling per pot 11 days after planting (DAP). Pots were set up in a completely randomized design inside the SPAR chambers, in 15 rows with 4 pots per row. Each carinata genotype was replicated five times within each temperature treatment. A total of 180 pots (12 genotypes × 3 treatments × 5 replications) were used in the study. Three temperature treatments (17/09°C – low, 22/14°C – optimum, and 29/19°C – high; day/night temperatures, respectively) were imposed 11 DAP, and plants were harvested 24 days after treatment (DAT) application.

**TABLE 1:** Details of *Brassica carinata* genotypes used in the study.

Genotype	Type^†^	Source/justification
AX17001	I	Selection from SE16-17 AYT (Avanza family selection) Florida
AX17002	I	Selection from SE16-17 AYT (Avanza family selection) Florida
AX17004	I	High shatter tolerance family, good potential in a winter environment
AX17005	I	High shatter tolerance family, good potential in a winter environment
AX17006	I	High shatter tolerance family, good potential in a winter environment
AX17007	DH	Among the highest Sclerotinia incidence, Jay and Quincy, FL
AX17008	DH	Selection from SE16-17 PYTB Florida
AX17009	DH	Selection from SE16-17 PYTA Florida
AX17010	DH	Selection from SE16-17 PYTB Florida
AX17014	H	Top 2016–2017 Quincy test hybrid Florida
AX17015	H	Promising test hybrid from 2017, frost tolerant female
Avanza 641	I	Commercial check

*^†^Genotypes are classified into three types (I, inbred; DH, double haploid; and H, hybrid). Seed trials (SE, Southeast; AYT, advanced yield trial; PYT, preliminary yield trial).*

### Measurements

#### Physiology Parameters

At 23 DAT, 1 day before final harvesting, physiological parameters, including chlorophyll (Chl), flavonoids (Flav), anthocyanin (Anth), and nitrogen balance index (NBI), were measured using a Dualex^®^ Scientific Polyphenols and Chlorophyll Meter (FORCE-A, Orsay, France). Additionally, a FluorPen FP 100 (Photon Systems Instruments, Drasov, Czech Republic) was used to collect the chlorophyll fluorescence (Fv′/Fm′). All measurements were collected from the second fully expanded leaf from the top of each plant.

#### Biomass Parameters

The shoot growth and developmental components for all 12 carinata genotypes evaluated included plant height, the total number of leaves, leaf area, leaf dry weight, stem dry weight, shoot weight, root dry weight, total dry weight, and root/shoot ratio (RS). The PH and LN were measured and counted 1 day before harvesting, and LA was recorded using an LI-3100 leaf-area meter (LI-COR, Inc., Lincoln, NE, United States). Leaves and stems were separated and dried in a forced-air oven at 75°C for 72 h, after which final dry biomass was recorded.

#### Root Parameters

At 24 DAT, all plants were harvested by separating the stem of each plant at ground level from its root system. Roots were then removed from the pots, placed on a wire screen, and washed thoroughly to remove the soil medium, using a moderate hydro flow speed and exercising maximum caution to avoid damage to the root structures. The longest root length was recorded using a meter ruler for each plant root. The individually cleaned root system was scanned using a Epson Expression 11000XL scanner, attached to a computer system. The individually cleaned root structures were placed onto a waterproof Plexiglas tray (40-cm length × 30-cm width) filled with approximately 5 mm of water and fitted onto the scanner. The roots were submerged, and the crossings and tips were spread using a small paintbrush to avoid overlapping. The acquired gray-scale root images were obtained through a high accuracy setting (resolution of 800 by 800 dpi) for the parameters measured by the WinRHIZO Pro 2009C software (Regent Instruments, Québec, Canada). The software calculated the following components: total root length, root surface area, average root diameter, root volume, number of root tips, forks, root crossings, root length density (ratio of total root length to root volume), and root to shoot percentage (ratio of root weight to shoot weight).

### Cumulative Low- and High-Temperature Response Index

Cumulative low-temperature response index (CLTRI) and cumulative high-temperature response index (CHTRI) values were calculated using the standardized vigor index (Wijewardana et al., 2015; Ramamoorthy et al., 2022). Initially, the individual stress response index (ISRI) for low temperature was calculated as the value of a parameter (P_*l*_) for a given genotype at the low temperature divided by the value of the same parameter at the optimum temperature (P_*o*_; Equation 1). Likewise, the ISRI for high temperature was calculated for each genotype as the parameter’s value at high temperature (P_*h*_) divided by the constant recorded for the same parameter at the optimum temperature (P_*o*_; Equation 2). The CLTRI (Equation 3) and CHTRI (Equation 4) were determined for each genotype by summing all the ISRI calculated for all the shoot and growth developmental, physiological, and root parameters measured across all genotypes. Trait acronyms and units are given in Table 2.


(1) $$\text{ISRI }\left(\text{low}\right)=P_{\text{l}}/P_{\text{o}}$$

(2) $$\text{ISRI }\left(\text{high}\right)=P_{\text{h}}/P_{\text{o}}$$

(3) CLTRI=(ChllChlo)+(FlavlFlavo)+(AnthlAntho)+(NBIlNBIo)+(Fv′/Fm′lFv′/Fm′o)+(PHlPHo)+(LAlLAo)+(LNlLNo)+(LWTlLWTo)+(SteWTlSteWTo)+(SWTlSWTo)+(TDMlTDMo)+(LRLlLRLo)+(TRLlTRLo)+(RSAlRSAo)+(RDlRDo)+(RVlRVo)+(RTlRTo)+(RFlRFo)+(RClRCo)+(RWTlRWTo)+(RLDlRLDo)+(RSlRSo)

(4) CHTRI=(ChlhChlo)+(FlavhFlavo)+(AnthhAntho)+(NBIhNBIo)+(Fv′/Fm′hFv′/Fm′o)+(PHhPHo)+(LAhLAo)+(LNhLNo)+(LWThLWTo)+(SteWThSteWTo)+(SWThSWTo)+(TDMhTDMo)+(LRLhLRLo)+(TRLhTRLo)+(RSAhRSAo)+(RDhRDo)+(RVhRVo)+(RThRTo)+(RFhRFo)+(RChRCo)+(RWThRWTo)+(RLDhRLDo)+(RShRSo)

**TABLE 2:** Summary of ANOVA across the genotype (G), temperature treatments (T), and their interaction (G × T) on a different shoot, root, and physiological traits measured 35 days after planting (24 days after temperature treatments imposition).

Trait	Unit	LT			HT			LT	OT	HT	Change (%)	
		G	T_*a*_	G × T_*a*_	G	T_*b*_	G × T_*b*_				LT	HT
**Physiology**												
Chlorophyll (Chl)	μg cm^–2^	*	ns	ns	**	ns	ns	20.5a	20.2a	20.6a	1.9	2.0
Flavonoids (Flav)	Unitless	ns	***	ns	**	***	ns	0.93a	0.68b	0.55c	36.9	−19.0
Anthocyanin (Anth)	Unitless	ns	**	ns	*	ns	ns	0.15a	0.14b	0.13b	7.9	−1.5
Nitrogen balance index (NBI)	Unitless	**	***	ns	***	***	ns	23.3c	31.1b	39.1a	−25.1	25.4
Chlorophyll fluorescence (Fv′/Fm′)	Unitless	ns	***	ns	ns	ns	ns	0.57b	0.66a	0.64a	−13.7	−3.3
**Shoot**												
Plant height (PH)	cm	***	***	*	***	**	ns	3.2c	9.8b	11.2a	−67.1	14.1
Leaf number (LN)	Number plant^–1^	**	***	ns	**	**	ns	3.3c	5.0b	5.5a	−34.8	9.3
Leaf area (LA)	cm^2^ plant^–1^	**	***	ns	***	ns	ns	265.3b	584.3a	595.4a	−54.6	1.9
Leaf weight (LWT)	g plant^–1^	**	***	ns	**	ns	ns	0.97b	1.89a	1.97a	−50.6	−3.7
Stem weight (SteWT)	g plant^–1^	**	***	ns	*	ns	ns	0.33b	0.91a	0.87a	−64.3	−4.7
Shoot weight (SWT)	g plant^–1^	**	***	ns	**	ns	ns	1.3b	2.9a	2.8a	−54.9	−4.0
Total dry matter (TDM)	g plant^–1^	*	***	ns	*	ns	ns	1.4b	3.1a	3.0a	−54.5	−4.3
**Root**												
Longest root length (LRL)	cm plant^–1^	ns	*	ns	ns	ns	ns	39.7b	44.7a	43.8ab	−11.1	−2.0
Total root length (TRL)	cm plant^–1^	ns	*	ns	ns	*	ns	1757.2b	2633.7a	1873.5b	−33.3	−28.9
Root surface area (RSA)	cm^2^ plant^–1^	ns	*	ns	ns	ns	ns	214.4b	314.2a	264.8ab	−31.8	−15.7
Root diameter (RD)	mm	ns	ns	ns	**	ns	ns	0.41a	0.40a	0.43a	2.1	9.1
Root volume (RV)	cm^3^	ns	*	ns	ns	ns	ns	2.1a	3.2a	3.1a	−32.9	−2.2
Root tips (RT)	Number plant^–1^	ns	***	ns	ns	ns	ns	4578b	9402.9a	9364.5a	−51.3	−0.4
Root forks (RF)	Number plant^–1^	ns	***	ns	ns	ns	ns	10195.3b	20264.8a	16458.5ab	−49.7	−18.8
Root crossings (RC)	Number plant^–1^	ns	***	ns	ns	**	ns	1205.2b	2333.4a	1426.1b	−48.4	−38.9
Root weight (RWT)	g plant^–1^	ns	***	ns	ns	ns	ns	0.13b	0.25a	0.23a	−48.7	−8.0
Root length density (RLD)	Ratio	*	*	ns	**	**	ns	819.8ab	909.3a	744.6b	−9.8	−18.1
Root to shoot ratio (RS)	Ratio	ns	**	ns	ns	ns	ns	0.09a	0.08b	0.07b	18.9	−6.3

Significance level: *p < 0.05, **p < 0.01, ***p < 0.001; ns, non-significant. LT, low temperature; OT, optimum temperature; and HT, high temperature. T_a_, control and low temperature; T_b_, control and high temperature treatment. Different letters indicate statistically significant least square difference (LSD) for treatments at the level of p < 0.05.

### Data Analysis

Phenotypic data were subjected to statistical analysis to determine the effect of temperature, genotype, and their interactions on the shoot, root, and physiological parameters using the library (‘‘doebioresearch’’) in RStudio 4.0.2.^1^ Least square difference (LSD) was used to compare the difference in the mean value between treatments or genotypes. Additionally, regression analysis was used to determine the relationship between temperature response indices and growth parameters among these response indices. Based on *r*^2^ values, best-fit regression functions were selected. Graphical analysis was done using Sigma Plot^®^ 14.5.

## Results and Discussion

Limited studies have phenotyped carinata genotypes for thermotolerance (low and high temperature) using different breed types (inbred, double haploid, and hybrid) at the early vegetative stage. Based on our knowledge, this is the first study to report variability in physiology, shoot and root morphological traits of advanced carinata genotypes to low- and high-temperature stresses during the early growth stage (Table 2). The information generated on carinata response to different temperatures will be beneficial for selecting genotypes for trait-based breeding programs.

### Physiological Parameters

**FIGURE 1:**
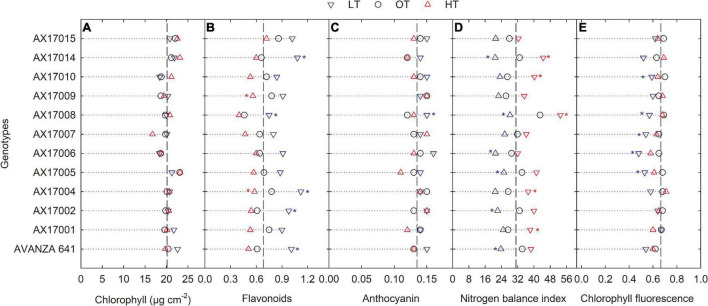
Line plots showing the phenotypic variations in physiological traits such as chlorophyll content **(A)**, flavonoids **(B)**, anthocyanin **(C)**, nitrogen balance index **(D)**, and chlorophyll fluorescence **(E)** of 12 advanced *Brassica carinata* genotypes measured 35 days after planting or 24 days after temperature treatments imposition. The dotted middle line indicates the average trait of 12 genotypes under optimum temperature. LT, low temperature; OT, optimum temperature; and HT, high temperature. Asterisk indicates a significant difference between the temperature treatments (blue – between LT and OT; red – between HT and OT) in a genotype at 5% LSD.

Low temperature affected the flavonoids, anthocyanin, NBI, and chlorophyll fluorescence (Fv′/Fm′) (*p* < 0.01, Table 2). The effect of treatment (low or high) was not significant for chlorophyll content (Table 2), which indicates the differential spread in response to treatment (Figure 1A). Low temperatures resulted in greater anthocyanin accumulation than at the optimum and high temperatures, but there was no difference between high and optimum temperatures (Table 2). The low temperature increased the accumulation of flavonoids (Figure 1B and Table 2) and anthocyanin (Figure 1C and Table 2). Five genotypes (AX17004, AX17014, AVANZA 641, AX17002, and AX17008) recorded significantly higher flavonoid values under low temperatures than the control (Figure 1B). At low temperature treatment, AX17008 recorded 25% (*p* < 0.05) higher anthocyanin than the optimum temperature (Figure 1C). The mean NBI decreased with decreasing temperature regimes by 25% (*p* < 0.05) from the optimum to the low temperature (Table 2). Across all treatments, genotype AX17008 had the greatest NBI (Figure 1D). Mean Fv′/Fm′ was not different between high temperature and optimum temperature treatments but decreased by 16% at the low temperature (Figure 1E). The Fv′/Fm′ was the least at low temperatures, and there was no difference between the optimum and high temperatures (Table 2). An increase in growing temperatures decreased leaves’ flavonoids and increased the NBI, indicating a negative relationship with flavonoids and a positive relationship with increased growing temperature (Figure 1). The effect of temperature on pigments indicates a strong thermal impact on the nitrogen status of carinata genotypes. Increased leaf flavonoid production can make plants resilient to environmental stresses by reducing oxidative stress damages (Kuk et al., 2003).

### Shoot Traits

**FIGURE 2:**
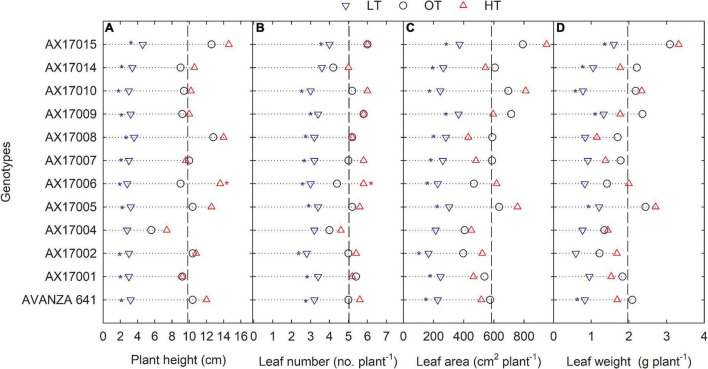
Line plots showing the phenotypic variations of shoot-related traits such as plant height **(A)**, number of leaves **(B)**, leaf area **(C)**, and leaf weight **(D)** of 12 advanced *Brassica carinata* genotypes measured 35 days after planting or 24 days after temperature treatments imposition. The dotted middle line indicates the average trait of 12 genotypes under optimum temperature. LT, low temperature; OT, optimum temperature; and HT, high temperature. Asterisk indicates a significant difference between the temperature treatments (blue – between LT and OT; red – between HT and OT) in a genotype at 5% LSD.

The genotypes significantly differed for plant height, leaf number, leaf weight, stem weight, and shoot weight (Table 2). Low temperature significantly decreased the plant height, leaf number, leaf weight, stem weight, and shoot weight for all genotypes by 67, 35, 55, 50, 64, and 60% at 24 days after stress (Table 2). In contrast, plant height, leaf number, and leaf weight were increased under high-temperature stress (Table 2). Two traits, PH and LN, had apparent differences among treatments ranking greatest to least from the high to low temperatures regimes (Figures 2A,B). The mean PH at the high temperature was 14% taller than the optimum temperature. The height reduction from optimum to low temperature was 67% (Figure 2A), showing the strong negative impact of low-temperature cell elongation and leaf expansion (Ben-Haj-Salah and Tardieu, 1995). Under the low temperature, a significantly lower number of leaves was observed in AX17002, while AX17006 grown under high temperature recorded a 51% greater number of leaves than at optimum temperature (Figure 2B). Under high temperatures, AX17015 had the tallest plants and the greatest LN, and AX17004 had the shortest plants (Figures 2A,B). Carinata stem elongation and leaf area expansion determine crop development and biomass accumulation in the early season. Across all the genotypes, shorter plants observed under low temperatures may be attributed to a reduction in cell division and elongation activities caused by low thermal conditions, affecting cellular functions and photosynthetic processes (Miedema, 1982; Ben-Haj-Salah and Tardieu, 1995). Under low temperature, the leaf area varied from 167 (AX17002) to 374 (AX17015), which is significantly lower than the other 2 treatments (Figure 2C). Under low temperature, all genotypes recorded a significant reduction in leaf area compared to optimum temperature treatment (Figure 2C). Leaf weight of 12 genotypes at the low temperature was lesser (55%) than at optimum temperature, but the response at high temperature was not different from the optimum (Figure 2D). The high temperature had no significant influence on the shoot biomass (leaf and stem weight, Table 2), but low-temperature treatment reduced the shoot weight by 55%. Likewise, studies of the same phenomena were noted in response to high temperatures in different crops (Wijewardana et al., 2015; Munyon et al., 2021; Reddy et al., 2021). The percentage of total dry matter reduction under high temperature was less than 5%, which was 50% lesser than the percentage reduction under low temperature (Table 2).

### Root Growth and Developmental Parameters

**FIGURE 3:**
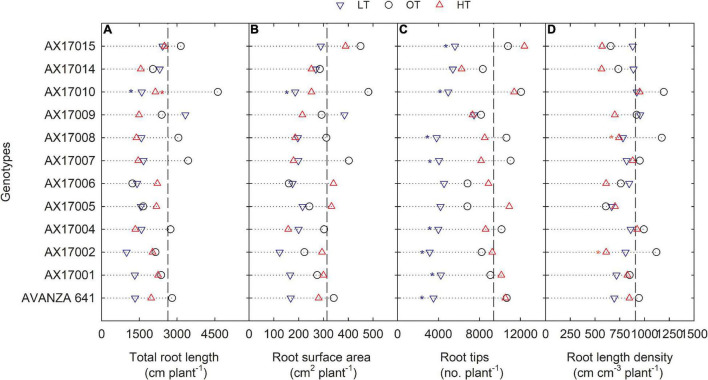
Line plots showing the phenotypic variations of root-related traits such as total root length **(A)**, root surface area **(B)**, root tips **(C)**, and root length density **(D)** of 12 advanced *Brassica carinata* genotypes measured 35 days after planting or 24 days after temperature treatments imposition. The dotted middle line indicates the average trait of 12 genotypes under optimum temperature. LT, low temperature; OT, optimum temperature; and HT, high temperature. Asterisk indicates a significant difference between the temperature treatments (blue – between LT and OT; red – between HT and OT) in a genotype at 5% LSD.

The effect of low temperature was significant (*p* < 0.05 to <0.001) on all the root parameters except root diameter. There were no temperature × genotype interactions (Table 2). The effect of high temperature was significant for total root length, root crossing, and root length density (Table 2). For all parameters except RD, trait values were highest at the optimum temperature (Table 2). Mean longest and total root length at the low temperature were 11% (*p* < 0.05) and 33% (*p* < 0.05) less than the optimum temperature, while at high temperature the same traits differed by 2 (*p* > 0.05) and 29% (*p* < 0.05) (Table 2). At low and high temperatures, AX17010 recorded a significantly lower total root length, 65 and 54% less than the total compared to the optimum temperature (Figure 3A). A reduction in plant root development under low temperatures may be due to its limited ability to access or uptake moisture and nutrient (Miedema, 1982). Suboptimal temperatures had similar damaging effects on root development in rice (Reddy et al., 2021) and cover crops (Munyon et al., 2021).

The root surface area of the 12 genotypes was 32% less at the low (214.4 cm^2^) than at the optimum temperature (314.2 cm^2^) (Table 2 and Figure 3B). The mean root volume of AX17010 was 57% less at the low temperature (2.1 cm^3^) compared to the optimum level (3.1 cm^3^), and the response at the high temperature was the same as the optimum (Figure 3C). This response suggests a more profound effect of low temperature on this root trait than the high temperature during the early growth stage. AX17009 showed no differences in root surface area, root tips, and root length density across treatments (Figure 3B). The mean number of root tips was reduced by 51% under low temperature than at the optimum, but the reduction was not significant between optimum and high temperature (Table 2 and Figure 3C). The average root fork of the 12 carinata genotypes decreased by 50% under low temperatures compared to the optimum temperature (Table 2). Mean root crossing among carinata genotypes was similar at low and high temperatures and was 48% less (low temperature) and 39% less (high temperature) compared to the optimum temperature (Table 2). The root length density (expressed as a total root length to volume) varied with treatment and genotypes (Table 2). In response to low and high temperatures, root length density decreased from 10% under low temperatures to 18% under high temperatures (Figure 3D). This study shows that low temperature inhibits most of the root traits’ development compared to optimum and high temperatures (Figure 3). This indicates that low thermal levels can restrict root growth and developmental processes due to a reduction in activities of enzymes related to membrane lipids of roots and decreased transport of photosynthetic products from shoots to the root system (Kaspar and Bland, 1992; Du and Tachibana, 1994; Arai-Sanoh et al., 2010).

### Biomass Production and Partitioning

**FIGURE 4:**
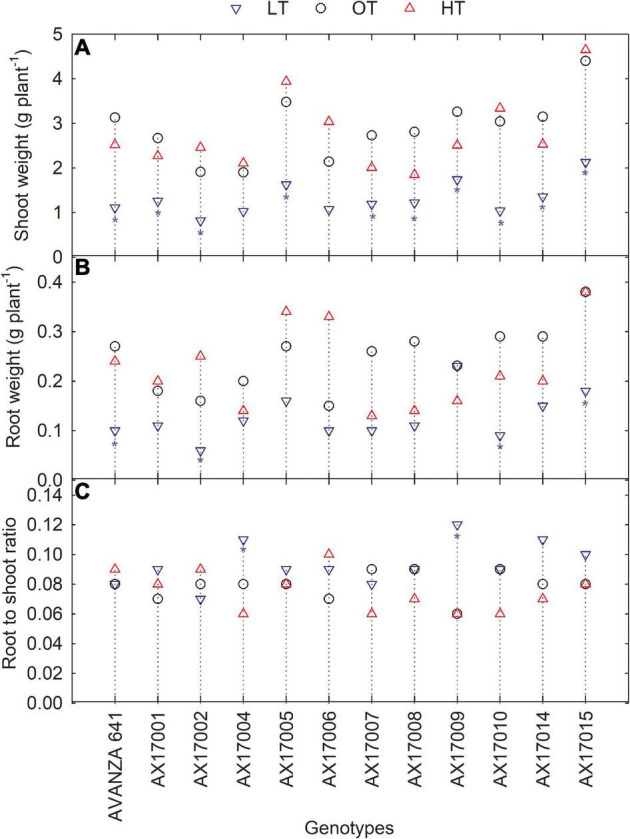
Line plots showing the phenotypic variations in shoot weight **(A)**, root weight **(B)**, and root to shoot ratio **(C)** of 12 carinata genotypes measured 35 days after planting or 24 days after temperature treatments imposition. The dotted middle line indicates the average trait of 12 genotypes under optimum temperature. LT, low temperature; OT, optimum temperature; and HT, high temperature. Asterisk indicates a significant difference between the temperature treatments (blue – between LT and OT; red – between HT and OT) in a genotype at 5% LSD.

Low temperature significantly affected the biomass (root and shoot) and root to shoot ratio (Table 2). While there was significant variation among genotypes for shoot weight, the high-temperature treatment had non-significant effects on the shoot and root weights (Table 2). Shoot weight decreased by 55% under low temperatures compared to the control (Figure 4A). Likewise, mean root weight across genotypes was also reduced by 49% (Figure 4B), indicating a thinner or shallow root system. Low temperature inhibited root component traits such as root tips, root crossing, root forks, and root surface (Table 2). Changes to the root to shoot ratio were significant, with a mean increase of 19% under low temperature compared to optimum temperature (Figure 4C). Conversely, root to shoot was substantially lower (6%) under high temperatures than the optimum temperature (Figure 4C). At the same time, all genotypes had no difference between high and optimum temperatures for root to shoot ratio. At low temperatures, AX17004 and AX17009 recorded significantly (*p* < 0.05) higher root to shoot ratios compared to the control (Figure 4C). The roots developed under low air or soil temperature were found to influence shoot biomass accumulation and resource allocation (Figure 4). These results show a stronger dependence on the physiology or metabolism of the shoot and root traits under stress conditions (Viana et al., 2022). Poor or weaker root growth and development during early crop establishment limit canopy growth and resource use efficiency at later crop stages (Moghimi et al., 2019; Nóia Júnior et al., 2022). In general, greater biomass allocation was recorded toward leaf (68, 63, and 63%) and stem (23, 29, and 29%) then to root (9, 8, and 7%) across (low, optimum, and high temperatures, respectively) treatments. Carinata shoot and root traits were more sensitive to low temperature than high-temperature stress (Table 1 and Figure 4).

Additionally, changes in traits’ response to low or high temperatures indicate that each trait or developmental event has its specific optimal temperature, which will decline above or below plant growth processes (Munyon et al., 2021). Our findings indicate that the short duration of high temperatures may not show a more significant impact on shoot traits. However, 24 days of high temperature was enough to induce changes in root formation, such as total root length and root length to density values. On the other hand, this study suggests the importance of future studies of carinata genotypes at different growth stages under gradient temperature conditions. As observed in our research, most growth traits had the most substantial growth and the developmental rate at the optimum temperature treatment. Although this was not tested under field conditions, carinata genotypes (AX17004 and AX17009) with higher tolerance to low temperature or chilling may be suitable for the southeastern United States climate.

#### Selection of Promising Low Temperature and High-Temperature Stress-Tolerant Carinata Genotypes

**FIGURE 5:**
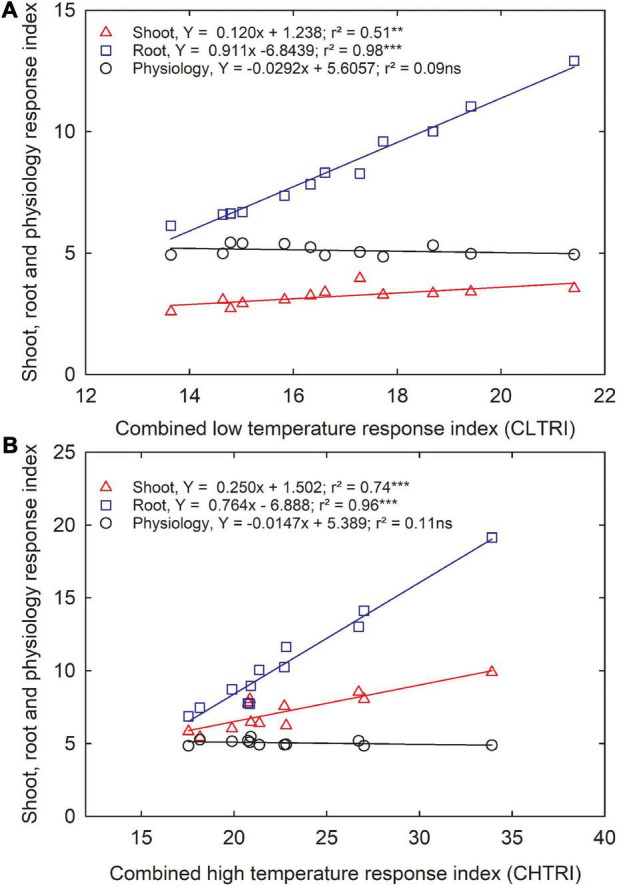
The relationship between shoot, root, and physiological response index and cumulative low-temperature response index (CLTRI, **A**) or cumulative high-temperature response index (CHTRI, **B**) of 12 advanced *Brassica carinata* genotypes was measured 35 days after planting or 24 days after temperature treatments. Significance level: **p < 0.01, ***p < 0.001; ns, non-significant.

Since information about carinata low- and high-temperature tolerance characteristics is unavailable, this study facilitates a better understanding of how the genotypes respond to low and high-temperature treatments at the early growth stage. The CLTRI and CHTRI were calculated to determine the relationship between shoot, root, and physiological components for the 12 advanced carinata genotypes grown under 3 temperature treatments during seedling growth and development. Under the low temperature, a strong relationship was observed between CLTRI and shoot (*r*^2^ = 0.51, *p* < 0.01) and root (*r*^2^ = 0.98, *p* < 0.001) components, indicating the importance of these two traits when selecting carinata genotypes for cold tolerance during the early vegetative growth stage (Figure 5A). A weak relationship (*r*^2^ = 0.07, *p* > 0.05) between physiological characteristics and CLTRI indicates greater sensitivity to low temperatures. Likewise, positive associations were observed between CHTRI and shoot (*r*^2^ = 0.74, *p* < 0.001) and root (*r*^2^ = 0.97, *p* < 0.001) components, which emphasized the dependence on the shoot and root development to improve stress tolerance of carinata genotypes during the early growth stage (Figure 5B). Also, a weak relationship was observed between CHTRI and the physiological traits (*r*^2^ = 0.10, *p* > 0.05; Figure 5B), indicating the sensitivity of physiological characteristics among the carinata genotypes. A weak linear relationship (*r*^2^ = 0.09, *p* > 0.05) between CLTRI and CHTRI suggests that genotype responses to low and high temperature are the same, indicating the presence of different stress tolerance mechanisms in carinata. Therefore, trait-based selection must be considered to improve low and high-temperature stress tolerance.

**FIGURE 6:**
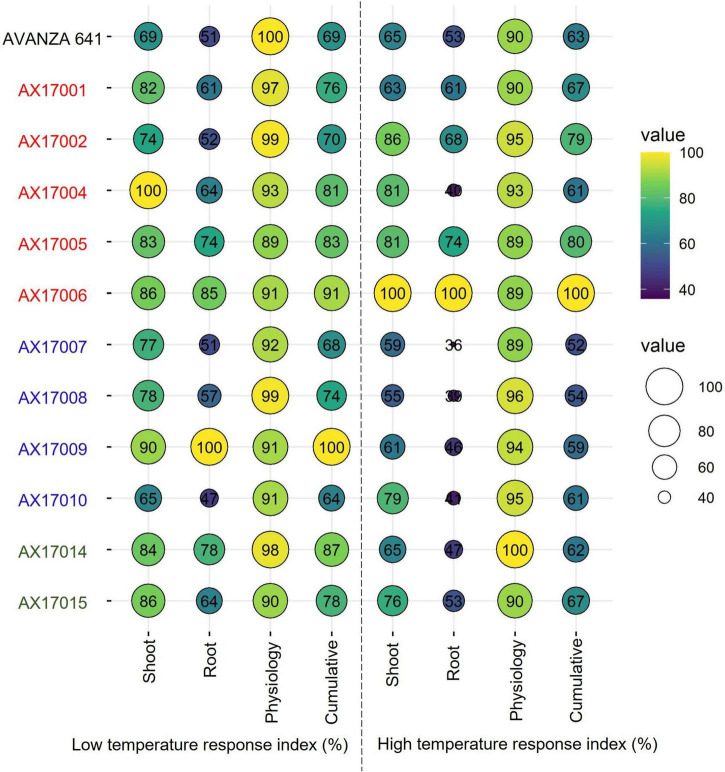
Bubble plot showing cumulative shoot, root, physiological, and cumulative low-temperature response index or high-temperature response index of 12 advanced *Brassica carinata* genotypes measured 35 days after planting or 24 days after temperature treatments. The colors of the genotypes represent four *Brassica carinata* breed types (*black* – cultivar or commercial check, *red* – inbred, *blue* – double haploid, and *green* – hybrid) used in the study.

Furthermore, individual genotype stress response index of the shoot, root, and physiological parameters or cumulative temperature response index was used to identify potential low- and high-temperature tolerant carinata genotypes (Figure 6), similar to other recent studies (Bheemanahalli et al., 2021; Reddy et al., 2021; Ramamoorthy et al., 2022). Among the genotypes studied, genotype AX17009 recorded a superior root system (no change in roots between low and optimum temperatures) coupled with shoot and physiology responses than the sensitive genotype (AX17010) at the early vegetative stage. The CHTRI percentage score varied between 52% (high temperature sensitive) and 100% (high temperature tolerant) among the genotypes (Figure 6). The genotype AX17006 was the highest temperature tolerant, while the genotypes such as AX17007 were highly heat-sensitive with CHTRI values less than 52% (Figure 6). The carinata genotype (AX17006) showed higher tolerance to low and high-temperature stresses based on the cumulative temperature response index. On the contrary, genotype AX17009 that top performed (high biomass) under low temperature became a weak performer under high temperature (Figure 6), indicating differential tolerance or adaptive to low and temperature stress at the early growth stage. When genotypes were grouped breed types (see color legend in Figure 6), a double haploid (AX17009) and an inbred (AX17006) had the maximum CLTRI than the commercial check (AVANZA 641). On average (relative scale), inbred genotypes (AX17001, AX17002, AX17005, and AX17006) exhibited substantially greater high-temperature tolerance to four double haploids (AX17007, AX17008, AX17009, and AX17010) at the early growth stage (Figure 6). Furthermore, given that this study was conducted under enclosed sunlit environmental conditions that mimic open field settings, these results could be transferred to natural field conditions (Allen et al., 2020), as was suggested in a similar study with cotton (Reddy et al., 1997). The data collected from this study will benefit future screening of carinata for low and high-temperature stress tolerance since it gives a more unambiguous indication of which traits are most relevant and should be considered when selecting for tolerance levels.

## Conclusion

Under low-temperature treatment, the 12 advanced carinata genotypes evaluated had substantial variability for the shoot, root, and physiological traits. Carinata genotypes are susceptible to low-temperature stress. The low temperature significantly limits various shoot traits, causing a 67, 34, 55, and 55% reduction in plant height, leaf number, leaf area, and total biomass. The suboptimal temperature had a higher impact on root formation traits such as root tips, root forks, and root crossings. Accordingly, total biomass was substantially reduced under low temperature, followed by high temperature compared to plants grown under optimum conditions. The maximum proportion of biomass partitioned to roots under low temperature than at the high-temperature stress, indicating the balance between the source and sink like growth and development. On a relative scale, the breed types used in the study showed differential tolerance to low and high temperatures at the early growth stage. Although we have not tested under field conditions, carinata genotypes (AX17004 and AX17009) with higher root to shoot ratios may be suitable for late-planting windows or regions with low-temperature spells. Further research is required to assess how carinata genotypes respond to low and high temperatures at later growth stages and in open field conditions. The heat- and cold-tolerant genotypes identified in this study would benefit plant breeders in developing genotypes adaptable to different climatic zones.

## Data Availability Statement

The original contributions presented in the study are included in the article/Supplementary Material, further inquiries can be directed to the corresponding author.

## Author Contributions

KR contributed to the conception and design of the work. LP and KR collected the data. RB edited the original draft and performed most of the writing, data analysis, and data visualization. LP, RS, KR, and BM review and editing. All authors contributed to the article’s critical revision.

## Conflict of Interest

The authors declare that the research was conducted in the absence of any commercial or financial relationships that could be construed as a potential conflict of interest.

## Publisher’s Note

All claims expressed in this article are solely those of the authors and do not necessarily represent those of their affiliated organizations, or those of the publisher, the editors and the reviewers. Any product that may be evaluated in this article, or claim that may be made by its manufacturer, is not guaranteed or endorsed by the publisher.

## Footnotes

1
https://www.rstudio.com/
