# Supplementary material for: Low- and High-Temperature Phenotypic Diversity of Brassica carinata Genotypes for Early-Season Growth and Development
Source: Front Plant Sci. 2022 Jun 14;13:900011. doi: 10.3389/fpls.2022.900011 (PMC9237576; doi:10.3389/fpls.2022.900011)
Supplement: Supplementary file 1 [file Data_Sheet_1.docx]

**Supplementary Table S1 |** Relative (ratio of low temperature to optimum temperature) responses of physiological, shoot, and root of twelve carinata genotypes to low temperature at 35 d after planting or 24 d after temperature treatment imposition.

| Trait | AVANZA641 | AX17001 | AX17002 | AX17004 | AX17005 | AX17006 | AX17007 | AX17008 | AX17009 | AX17010 | AX17014 | AX17015 |
| --- | --- | --- | --- | --- | --- | --- | --- | --- | --- | --- | --- | --- |
| Chl | 1.11 | 1.11 | 1.03 | 1.03 | 0.92 | 0.99 | 1.02 | 1.01 | 1.09 | 0.98 | 1.03 | 0.94 |
| Flav | 1.65 | 1.20 | 1.60 | 1.44 | 1.28 | 1.41 | 1.26 | 1.64 | 1.17 | 1.17 | 1.64 | 1.20 |
| Anth | 1.12 | 1.02 | 1.15 | 0.95 | 1.13 | 1.11 | 1.07 | 1.25 | 0.89 | 1.07 | 1.18 | 1.11 |
| NBI | 0.69 | 0.91 | 0.67 | 0.77 | 0.74 | 0.73 | 0.80 | 0.66 | 0.86 | 0.86 | 0.64 | 0.76 |
| Fv/Fm | 0.87 | 1.00 | 0.94 | 0.85 | 0.78 | 0.74 | 0.83 | 0.83 | 0.93 | 0.84 | 0.83 | 0.90 |
| PH | 0.31 | 0.33 | 0.29 | 0.50 | 0.31 | 0.31 | 0.30 | 0.28 | 0.35 | 0.32 | 0.38 | 0.37 |
| LN | 0.64 | 0.63 | 0.56 | 0.80 | 0.65 | 0.68 | 0.64 | 0.62 | 0.59 | 0.58 | 0.86 | 0.67 |
| LA | 0.40 | 0.46 | 0.42 | 0.53 | 0.48 | 0.49 | 0.45 | 0.48 | 0.51 | 0.35 | 0.44 | 0.47 |
| LWT | 0.39 | 0.52 | 0.49 | 0.57 | 0.50 | 0.58 | 0.51 | 0.49 | 0.57 | 0.36 | 0.48 | 0.52 |
| SteWT | 0.27 | 0.37 | 0.32 | 0.48 | 0.40 | 0.34 | 0.30 | 0.34 | 0.44 | 0.30 | 0.32 | 0.41 |
| SWT | 0.35 | 0.47 | 0.43 | 0.54 | 0.47 | 0.50 | 0.44 | 0.44 | 0.53 | 0.34 | 0.43 | 0.48 |
| TDM | 0.36 | 0.48 | 0.43 | 0.55 | 0.48 | 0.51 | 0.43 | 0.43 | 0.56 | 0.34 | 0.44 | 0.48 |
| LRL | 0.79 | 1.01 | 0.74 | 1.06 | 0.92 | 0.83 | 0.83 | 0.92 | 0.97 | 0.94 | 0.87 | 0.84 |
| RL | 0.47 | 0.56 | 0.46 | 0.58 | 0.94 | 1.15 | 0.48 | 0.52 | 1.39 | 0.35 | 1.13 | 0.77 |
| RSA | 0.49 | 0.60 | 0.55 | 0.66 | 0.89 | 1.09 | 0.49 | 0.63 | 1.32 | 0.39 | 0.94 | 0.64 |
| RD | 1.12 | 1.12 | 1.16 | 1.13 | 0.96 | 0.95 | 1.04 | 1.25 | 0.97 | 1.15 | 0.79 | 0.81 |
| RV | 0.50 | 0.66 | 0.66 | 0.78 | 0.83 | 1.04 | 0.49 | 0.78 | 1.24 | 0.43 | 0.67 | 0.49 |
| RT | 0.33 | 0.47 | 0.38 | 0.39 | 0.62 | 0.66 | 0.37 | 0.36 | 0.92 | 0.41 | 0.65 | 0.52 |
| RF | 0.35 | 0.38 | 0.37 | 0.42 | 0.72 | 1.09 | 0.37 | 0.44 | 0.96 | 0.21 | 0.80 | 0.55 |
| RC | 0.40 | 0.38 | 0.29 | 0.40 | 0.86 | 1.23 | 0.36 | 0.38 | 1.10 | 0.21 | 1.01 | 0.66 |
| RWT | 0.36 | 0.58 | 0.39 | 0.60 | 0.58 | 0.64 | 0.40 | 0.41 | 1.00 | 0.31 | 0.53 | 0.48 |
| RLD | 0.74 | 0.84 | 0.72 | 0.87 | 1.10 | 1.11 | 0.86 | 0.67 | 1.04 | 0.77 | 1.21 | 1.34 |
| RS | 1.08 | 1.23 | 0.97 | 1.39 | 1.17 | 1.24 | 0.89 | 1.01 | 2.02 | 0.96 | 1.42 | 1.20 |

Chlorophyll (Chl), Flavonoids (Flav), Anthocyanin (Anth), Nitrogen balance index (NBI), Chlorophyll fluorescence (Fv’/Fm’), Plant height (PH), Leaf number (LN), Leaf area (LA), Leaf weight (LWT), Stem weight (SteWT), Shoot weight (SWT), Total dry matter (TDM), Longest root length (LRL), Total root length (TRL), Root surface area (RSA), Root diameter (RD), Root volume (RV), Root tips (RT), Root forks (RF), Root crossings (RC), Root weight (RWT), Root length density (RLD), and Root to shoot ratio (RS).

**Supplementary Table S2 |** Relative (ratio of high temperature to optimum temperature) responses of physiological, shoot, and root of twelve carinata genotypes to high temperature at 35 d after planting or 24 d after temperature treatment imposition.

| Trait | AVANZA641 | AX17001 | AX17002 | AX17004 | AX17005 | AX17006 | AX17007 | AX17008 | AX17009 | AX17010 | AX17014 | AX17015 |
| --- | --- | --- | --- | --- | --- | --- | --- | --- | --- | --- | --- | --- |
| Chl | 0.97 | 1.03 | 1.04 | 1.03 | 1.00 | 1.01 | 0.85 | 1.05 | 1.04 | 1.13 | 1.09 | 1.02 |
| Flav | 0.84 | 0.71 | 0.88 | 0.74 | 0.83 | 0.93 | 0.74 | 0.88 | 0.73 | 0.74 | 0.91 | 0.85 |
| Anth | 1.01 | 0.88 | 1.08 | 0.91 | 0.91 | 0.94 | 1.16 | 1.11 | 0.99 | 0.90 | 1.01 | 0.96 |
| NBI | 1.12 | 1.40 | 1.22 | 1.36 | 1.21 | 1.11 | 1.14 | 1.23 | 1.34 | 1.49 | 1.35 | 1.16 |
| Fv/Fm | 0.97 | 0.91 | 0.96 | 1.04 | 0.90 | 0.89 | 0.96 | 0.98 | 1.05 | 0.92 | 1.09 | 0.93 |
| PH | 1.15 | 1.00 | 1.04 | 1.32 | 1.21 | 1.51 | 0.96 | 1.09 | 1.09 | 1.09 | 1.18 | 1.16 |
| LN | 1.12 | 0.96 | 1.08 | 1.15 | 1.08 | 1.32 | 1.16 | 1.00 | 1.00 | 1.15 | 1.19 | 1.00 |
| LA | 0.90 | 0.86 | 1.32 | 1.11 | 1.19 | 1.32 | 0.82 | 0.73 | 0.83 | 1.16 | 0.90 | 1.20 |
| LWT | 0.81 | 0.84 | 1.38 | 1.06 | 1.11 | 1.42 | 0.77 | 0.68 | 0.75 | 1.07 | 0.80 | 1.08 |
| SteWT | 0.80 | 0.87 | 1.13 | 1.22 | 1.18 | 1.43 | 0.67 | 0.63 | 0.82 | 1.16 | 0.81 | 1.01 |
| SWT | 0.81 | 0.85 | 1.29 | 1.11 | 1.13 | 1.42 | 0.74 | 0.66 | 0.77 | 1.10 | 0.80 | 1.06 |
| TDM | 0.81 | 0.86 | 1.31 | 1.07 | 1.14 | 1.47 | 0.72 | 0.65 | 0.77 | 1.07 | 0.79 | 1.05 |
| LRL | 0.99 | 0.97 | 0.88 | 1.17 | 0.99 | 1.15 | 1.01 | 0.96 | 0.86 | 1.00 | 0.85 | 1.00 |
| RL | 0.70 | 0.95 | 0.94 | 0.49 | 1.32 | 1.81 | 0.42 | 0.45 | 0.62 | 0.46 | 0.76 | 0.79 |
| RSA | 0.82 | 1.09 | 1.32 | 0.52 | 1.37 | 2.12 | 0.45 | 0.60 | 0.73 | 0.52 | 0.88 | 0.87 |
| RD | 1.09 | 1.04 | 1.36 | 1.03 | 0.97 | 1.14 | 1.00 | 1.28 | 1.18 | 1.12 | 1.02 | 0.99 |
| RV | 0.98 | 1.27 | 1.83 | 0.55 | 1.47 | 2.50 | 0.46 | 0.80 | 0.89 | 0.59 | 0.89 | 0.87 |
| RT | 0.99 | 1.12 | 1.13 | 0.85 | 1.60 | 1.30 | 0.74 | 0.80 | 0.90 | 0.94 | 0.75 | 1.15 |
| RF | 0.84 | 1.07 | 1.52 | 0.40 | 1.52 | 2.57 | 0.36 | 0.46 | 0.64 | 0.53 | 0.85 | 0.88 |
| RC | 0.71 | 0.82 | 0.82 | 0.32 | 1.45 | 2.31 | 0.30 | 0.27 | 0.51 | 0.39 | 0.66 | 0.80 |
| RWT | 0.89 | 1.11 | 1.57 | 0.69 | 1.25 | 2.12 | 0.52 | 0.49 | 0.72 | 0.73 | 0.68 | 1.01 |
| RLD | 0.90 | 0.97 | 0.55 | 0.93 | 1.15 | 0.80 | 0.92 | 0.63 | 0.76 | 0.80 | 0.77 | 0.87 |
| RS | 1.15 | 1.22 | 1.10 | 0.77 | 1.05 | 1.32 | 0.69 | 0.71 | 0.92 | 0.67 | 0.84 | 1.02 |

Chlorophyll (Chl), Flavonoids (Flav), Anthocyanin (Anth), Nitrogen balance index (NBI), Chlorophyll fluorescence (Fv’/Fm’), Plant height (PH), Leaf number (LN), Leaf area (LA), Leaf weight (LWT), Stem weight (SteWT), Shoot weight (SWT), Total dry matter (TDM), Longest root length (LRL), Total root length (TRL), Root surface area (RSA), Root diameter (RD), Root volume (RV), Root tips (RT), Root forks (RF), Root crossings (RC), Root weight (RWT), Root length density (RLD), and Root to shoot ratio (RS).
